# Hesperidin-3′-*O*-Methylether Is More Potent than Hesperidin in Phosphodiesterase Inhibition and Suppression of Ovalbumin-Induced Airway Hyperresponsiveness

**DOI:** 10.1155/2012/908562

**Published:** 2012-10-03

**Authors:** You-Lan Yang, Hsin-Te Hsu, Kuo-Hsien Wang, Chao-Sian Wang, Chien-Ming Chen, Wun-Chang Ko

**Affiliations:** ^1^School of Respiratory Therapy, College of Medicine, Taipei Medical University, Taipei 110, Taiwan; ^2^Department of Otolaryngology, Taipei Medical University Hospital, Taipei 110, Taiwan; ^3^Department of Dermatology, Taipei Medical University Hospital, Taipei 110, Taiwan; ^4^Department of Pharmacology, College of Medicine, Taipei Medical University, Taipei 110, Taiwan; ^5^Department of Medical Technology, College of Medicine, Taipei Medical University, Taipei 110, Taiwan

## Abstract

Hesperidin is present in the traditional Chinese medicine, “Chen Pi,” and recently was reported to have anti-inflammatory effects. Therefore, we were interested in comparing the effects of hesperidin and hesperidin-3′-*O*-methylether on phosphodiesterase inhibition and airway hyperresponsiveness (AHR) in a murine model of asthma. In the present results, hesperidin-3′-*O*-methylether, but not hesperidin, at 30 **μ**mol/kg (p.o.) significantly attenuated the enhanced pause (*P*_enh_) value, suppressed the increases in numbers of total inflammatory cells, macrophages, lymphocytes, neutrophils, and eosinophils, suppressed total and OVA-specific immunoglobulin (Ig)E levels in the serum and BALF, and enhanced the level of total IgG_2a_ in the serum of sensitized and challenged mice, suggesting that hesperidin-3′-*O*-methylether is more potent than hesperidin in suppression of AHR and immunoregulation. The different potency between them may be due to their aglycons, because these two flavanone glycosides should be hydrolyzed by **β**-glucosidase after oral administration. Neither influenced xylazine/ketamine-induced anesthesia, suggesting that they may have few or no adverse effects, such as nausea, vomiting, and gastric hypersecretion. In conclusion, hesperidin-3′-*O*-methylether is more potent in phosphodiesterase inhibition and suppression of AHR and has higher therapeutic (PDE4_*H*_/PDE4_*L*_) ratio than hesperidin. Thus, hesperidin-3′-*O*-methylether may have more potential for use in treating allergic asthma and chronic obstructive pulmonary disease.

## 1. Introduction

Phosphodiesterases (PDEs) are classified according to their primary protein and complementary (c)DNA sequences, co-factors, substrate specificities, and pharmacological roles. It is now known that phosphodiesterases (PDEs) comprise at least 11 distinct enzyme families that hydrolyze adenosine 3′,5′ cyclic monophosphate (cAMP) and/or guanosine 3′,5′ cyclic monophosphate (cGMP) [1]. PDE1*∼*5 isozymes, which are calcium/calmodulin dependent (PDE1), cGMP stimulated (PDE2), cGMP inhibited (PDE3), cAMP specific (PDE4), and cGMP specific (PDE5), were found to be present in the canine trachea [2], guinea pig lungs [3], and human bronchi [4]. PDE3 and PDE4 were identified in the guinea pig airway [5], but other isozymes might also be present. PDE4 may adopt two different conformations which have high (PDE4_*H*_) and low (PDE4_*L*_) affinities for rolipram, respectively. It is believed that inhibition of PDE4_*H*_ is associated with adverse responses, such as nausea, vomiting, and gastric hypersecretion, while inhibition of PDE4_*L*_ is associated with anti-inflammatory and bronchodilating effects. Therefore the therapeutic ratio of selective PDE4 inhibitors for use in treating asthma and chronic obstructive pulmonary disease (COPD) is defined as the PDE4_*H*_/PDE4_*L*_ ratio [6, 7]. Although both asthma and COPD are associated with an underlying chronic inflammation of the airways, there are important differences with regard to the inflammatory cells and mediators involved. The key inflammatory cells in COPD are macrophages, CD8+ T-lymphocytes, and neutrophils. In contrast, the key inflammatory cells in asthma are mast cells, eosinophils, and CD4+ T-lymphocytes. Both diseases are sensitive to steroids. However, COPD shows a limited response to inhaled corticosteroids as compared to the efficacy achieved in asthma. Other therapeutic drugs such as selective PDE4 or dual PDE3/4 inhibitors are recently developing. However, these developing inhibitors are also limited for the use of asthma and COPD in clinic because of their emetic side effect. This side effect can be easily assessed in nonvomiting species, such as rats or mice, in which selective PDE4 inhibitors reduce the duration of xylazine/ketamine-induced anesthesia [8, 9].

Hesperetin, one of the most-common flavonoids in *Citrus*, was reported to selectively inhibit PDE4 activity [10]. Men with higher hesperetin intake have lower mortality from cerebrovascular disease and lung cancer, and lower incidences of asthma [11]. Hesperetin frequently occurs in nature as glycosides, such as hesperidin and neohesperidin. They are abundantly present in the fruit peel of *Citrus aurantium* L. (Rutaceae), a well-known traditional Chinese medicine called “Chen-Pi”, which is used as an expectorant and stomach tonic, and contains vitamin P, a remedy for preventing capillary fragility and hypertension [12]. These glycosides are easily hydrolyzed by glycosidase to form hesperetin after ingestion. Hesperidin was recently reported to inhibit inflammatory cell infiltration and mucus hypersecretion in a murine model of asthma [13]. Therefore, we were interested in comparing the effects of hesperidin and hesperidin-3′-*O*-methylether, a more-liposoluble derivative of hesperidin, on PDE1*∼*5 inhibition and suppression on ovalbumin-induced airway hyperresponsiveness (AHR). To clarify their potentials for use in treating asthma and COPD, their PDE4_*H*_/PDE4_*L*_ ratios were also investigated.

## 2. Materials and Methods

### 2.1. Reagents and Animals

**Figure 1:**
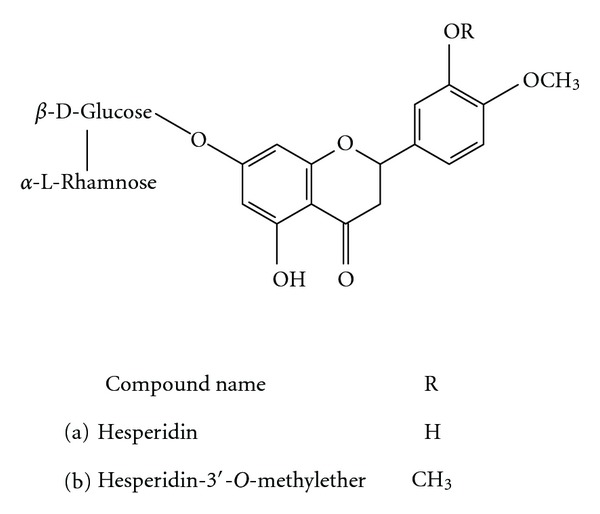
The structures of hesperidin (a) and hesperidin-3′-*O*-methylether (b).

Hesperidin (mol wt., 610.60) and hesperidin-3′-*O*-methylether (mol wt., 624.59) were purchased from Sigma Chemical (St. Louis, MO, USA) and Tokyo Chemical Industry (Tokyo, Japan), respectively. Their structures are shown in Figure 1. From Sigma Chemical, ovalbumin, methacholine, aluminum sulfate hexadecahydrate, dimethylsulfoxide (DMSO), chloralose, urethane, Tris-HCl, Bis-Tris, benzamidine, phenylmethanesulfonyl fluoride (PMSF), *d,l*-dithiothreitol, polyethyleneimine, ethylenediaminetetraacetic acid (EDTA), bovine serum albumin (BSA), cAMP, cGMP, calmodulin, Dowex resin, *Crotalus atrox *snake venom, xylazine, and ketamine were also purchased. Vinpocetine, *erythro*-9-(2-hydroxy-3-nonyl)-adenine HCl (EHNA), milrinone, 4-(3-butoxy-4-methoxybenzyl)-2-imidazolidinone (Ro 20-1724), and zaprinast were purchased from Biomol (Plymouth Meeting, PA, USA). Mouse T helper (Th)1/Th2 cytokine CBA kits and mouse IgE enzyme-linked immunosorbent assay (ELISA) sets were purchased from Pharmingen (San Diego, CA, USA). Ethyl alcohol and polyethylene glycol (PEG) 400 were purchased from Merck (Darmstadt, Germany). [^3^ *H*]-cAMP, [^3^ *H*]-cGMP, and [*methyl*-^3^ *H*]-rolipram were purchased from Amersham Pharmacia Biotech (Buckinghamshire, UK). Other reagents, such as CaCl_2_, MgCl_2_, and NaCl, were of analytical grade. Hesperidin, hesperidin-3′-*O*-methylether, milrinone, Ro 20-1724, and zaprinast were dissolved in DMSO. Vinpocetine, EHNA, and PMSF were dissolved in 95% ethyl alcohol. Other reagents were dissolved in distilled water.

Male Dunkin Hartley guinea pigs (500*∼*600 g), and female BABL/c mice at 8*∼*12 weeks old were purchased from the Animal Center of the National Science Council (Taipei, Taiwan), and housed in ordinary cages at 22 ± 1°C with a humidity of 50%*∼*60% under a constant 12/12-h light/dark cycle and provided with food and water *ad libitum*. Under a protocol approved by the Animal Care and Use Committee of Taipei Medical University, the following *in vivo* and *in vitro* experiments were performed.

### 2.2. Inhibition of PDE1, PDE3, and PDE4 Activities by Hesperidin-3′-*O*-Methylether

Activities of PDE1*∼*5 in the homogenate of guinea pig lungs or hearts [14] were measured by a two-step procedure according to the previous method [15], using cAMP with [^3^ *H*]-cAMP or cGMP with [^3^ *H*]-cGMP as substrates. The enzyme preparation (25 *μ*L) was incubated for 30 min at 37°C in a total assay volume of 100 *μ*L containing 50 mM Tris-HCl (pH 7.4), 3 mM MgCl_2_, 1 mM dithiothreitol, 0.05% BSA, and 1 *μ*M cAMP with 0.2 *μ*Ci [^3^ *H*]-cAMP as a substrate alone or in the presence of 0.1 unit calmodulin with 10 *μ*M CaCl_2_ or 5 *μ*M cGMP, and 1 *μ*M cGMP with 0.2 *μ*Ci [^3^ *H*]-cGMP as another substrate alone or in the presence of 0.1 unit calmodulin with 10 *μ*M CaCl_2_. The reaction mixture contained 10 *μ*L of vehicle or test compounds including hesperidin, hesperidin-3′-*O*-methylether, or selective PDE1*∼*5 inhibitors, such as vinpocetine [16], EHNA [17], milrinone [18], Ro 20-1724 [19], and zaprinast [20] as reference drugs. The reagents and homogenate were mixed on ice, and the reaction was initiated by transferring the mixture to a water bath at 37°C. Following a 30 min incubation, the reaction was stopped by transferring the reaction vessel to a bath of boiling water for 3 min. After cooling on ice, 20 *μ*L of a 1 mg/mL solution of *Crotalus atrox* snake venom was added to the reaction mixture, and the mixture was incubated at 37°C for 10 min. Unreacted [^3^ *H*]-cAMP or [^3^ *H*]-cGMP was removed by the addition of 500 *μ*L of a 1-in-1 Tris-HCl (40 mM) buffer suspension of Dowex resin (1 × 2-400) with incubation on ice for 30 min. Each tube was then centrifuged at 3700 g for 2 min, and 100 *μ*L of the supernatant was removed for liquid scintillation counting. Less than 10% of the tritiated cyclic nucleotide was hydrolyzed in this assay.

### 2.3. Determination of PDE4_*H*_ Values

When the above-mentioned guinea pigs were sacrificed, the whole brains were removed and homogenized with a glass/Teflon homogenizer (Glas-Col, Terre Haute, IN, USA) in 10 volumes of cold medium (pH 6.5) containing 20 mM Bis-Tris, 2 mM benzamidine, 2 mM EDTA, 50 mM sodium chloride, 0.1 mM PMSF, and 1 mM dithiothreitol. At 4°C, the homogenate was centrifuged at 170 g for 5 min to remove connective tissues and blood vessels. The suspended homogenate was then recentrifuged at 40,000 g for 30 min to separate the cytosolic and particulate portions. The particulate portion was resuspended in a suspension at a concentration of 400 mg/mL (wet weight/volume), after washing three times with homogenizing buffer. The particulate portion mainly consisted of cell membranes. The binding ability of hesperidin (300 *μ*M) or hesperidin-3′-*O*-methylether (3*∼*300 *μ*M) to high-affinity rolipram-binding sites (HARBSs) of membranes was determined by replacing 2 nM [^3^ *H*]-rolipram in a reaction buffer at 30°C for 1 h, according to the method described by previous investigators [21, 22] and modified by us. Briefly, the reaction buffer consisted of 50 mM Tris-HCl and 5 mM MgCl_2_ (pH 7.5). The total volume of the reaction mixture was 25 *μ*L, consisting of 10 *μ*L of the particulate suspension, 10 *μ*L of [^3^ *H*]-rolipram, and 5 *μ*L of hesperidin, hesperidin-3′-*O*-methylether, or reference drug, such as Ro 20-1724 (1*∼*10,000 nM). After 1 h, the reaction was terminated by moving the reaction vessel into crushed ice. Then the reaction mixture was transferred onto Whatman GF/B glass-fiber filters, which were soaked in a 0.3% polyethyleneimine solution in a mini-funnel. The reaction mixture was filtered by centrifugation at 90 g for 10 s, and the filtrate was collected into a 1.5-mL Eppendorf tube with the top adapted to the outlet of the mini-funnel. The filters were washed with 300 *μ*L of the reaction buffer three times each in the same way and transferred into 2 mL of cocktail for radiation counting (total binding) using a *β*-scintillation counter (Beckman, Fullerton, CA, USA). Nonspecific binding, which was defined in the presence of 10 *μ*M Ro 20-1724, was subtracted from total binding to yield specific binding. Effective concentration (EC_50_) values of hesperidin, hesperidin-3′-*O*-methylether, and Ro 20-1724, at which a half of the [^3^ *H*]-rolipram that was bound onto HARBSs of cell membranes was displaced, were defined as PDE4_*H*_  values, and these were related to any adverse effects, such as nausea, vomiting, and gastric hypersecretion [7].

### 2.4. AHR *In Vivo*

**Figure 2:**
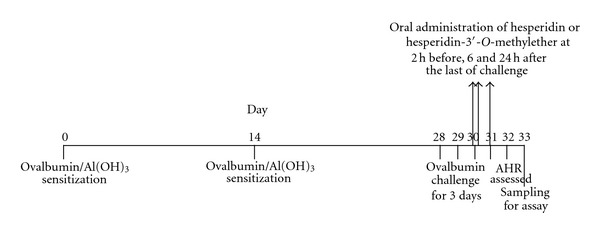
The schedule of sensitization, challenge, and drug administration in mice. AHR airway hyperresponsiveness; Al(OH)_3_: aluminum hydroxide gel.

According to the schedule (Figure 2), ten female BABL/c mice in each group were sensitized by an intraperitoneal (i.p.) injection of 20 *μ*g of ovalbumin emulsified in 2.25 mg of an aluminum hydroxide gel, prepared from aluminum sulfate hexadecahydrate, in a total volume of 100 *μ*L on days 0 and 14. Mice were challenged *via* the airway using 1% ovalbumin in saline for 30 min on days 28, 29, and 30 by ultrasonic nebulization. After the last ovalbumin challenge [23], AHR was assessed on day 32 (48 h after 1% ovalbumin provocation) in each group. Each group of mice was orally (p.o.) administered the vehicle (control), 30*∼*100 *μ*mol/kg of hesperidin or 10*∼*100 *μ*mol/kg of hesperidin-3′-*O*-methylether 2 h before and 6 and 24 h after ovalbumin provocation. For comparison, sham-treated mice were challenged with saline instead of 1% ovalbumin (nonchallenged). The vehicle, a mixture of DMSO : PEG 400 : saline (1 : 1 : 8, v/v), hesperidin, or hesperidin-3′-*O*-methylether was administered (p.o.) at a volume of 0.01 mL/g of body weight. AHR was assessed by barometric plethysmography [24] using a whole-body plethysmograph (WBP) and analyzed using software of Life Science Suite P3 Analysis Modules (Gould, LDS Test and Measurement LLC, Valley View, OH, USA) in unrestrained animals. Mice were placed into the main chamber of the WBP, and the baseline enhanced pause (*P*_enh_) value was determined. Then mice were first nebulized with phosphate-buffered saline (PBS), and subsequently with increasing doses (6.25*∼*50 mg/mL) of methacholine for 3 min for each nebulization, followed by readings of breathing parameters for 3 min after each nebulization to determine *P*_enh_ values. Twenty-four hours after *P*_enh_ determination, these mice were anesthetized with pentobarbital (50 mg/kg, i.p.), and the lungs were lavaged *via* a tracheal tube with PBS (1 × 1.0 mL, 37°C). After lavage, blood was collected from the jugular vein and allowed to sit so that it would coagulate. The collected bronchoalveolar lavage fluid (BALF) and coagulated blood were respectively centrifuged at 630 g for 7 min and at 3700 g for 10 min at 4°C. After centrifugation, the BALF and serum supernatants were stored at −20°C until determinations of cytokines, including interleukin (IL)-2, IL-4, IL-5, tumor necrosis factor (TNF)-*α*, and interferon (IFN)-*γ* by flow cytometric methods [25] using mouse Th1/Th2 cytokine CBA kits, and of a total immunoglobulin (Ig)E and IgG_2a_ using ELISA kits (Pharmingen, San Diego, CA, USA) according to the respective recommendations of the manufacturers. Ovalbumin-specific IgE was measured as described previously [26]. Wells were coated with 100 *μ*L of ovalbumin (20 *μ*g/mL) instead of the capture antibody. Levels are expressed in arbitrary units, where 1 arbitrary unit equals the optical density of the sample divided by the optical density of unchallenged mouse serum or BALF (standard). The BALF pellet was resuspended in ACK lysing buffer (1.658 g NH_4_Cl, 0.2 g KHCO_3_, and 1.44 mg EDTA in 200 mL of water) to lyse the residual erythrocytes in each sample. The number of inflammatory cells was counted using a hemocytometer (Hausser Scientific, Horsham, PA, USA). Cytospin slides were stained and cell differentials were determined in a blinded fashion by counting at least 100 cells under light microscopy. All undetectable data (<1 pg/mL) of cytokines were taken as 0.5 pg/mL.

### 2.5. Xylazine/Ketamine-Induced Anesthesia

According to a previously described method [9] and modified by us, hesperidin, hesperidin-3′-*O*-methylether (each 300 *μ*mol/kg, subcutaneously (s.c.)), or Ro 20-1724 (0.01*∼*1 *μ*mol/kg, s.c.), a reference drug, was, respectively, injected into 8*∼*12-week-old female BALB/c mice 1, 1 or 0.25 h prior to an i.p. injection of xylazine (10 mg/kg)/ketamine (70 mg/kg). The vehicle (control) for hesperidin, hesperidin-3′-*O*-methylether, or Ro 20-1724 was a mixture of DMSO : PEG 400 : saline (1 : 1 : 8, v/v). After the loss of the righting reflex (i.e., when a mouse remained on its back and no longer spontaneously righted itself to a prone position), the duration of anesthesia was measured until its return as the endpoint [9].

### 2.6. Statistical Analysis

All values are given as the mean ± SEM. Differences among values were statistically calculated by one-way analysis of variance (ANOVA), and then determined by Dunnett's test. The difference between two values, however, was determined by the use of Student's *t*-test. Differences with *P* < 0.05 were considered statistically significant.

## 3. Results

### 3.1. Inhibition of PDE1, PDE3, and PDE4 Activities by Hesperidin-3′-O-methylether

**Figure 3:**
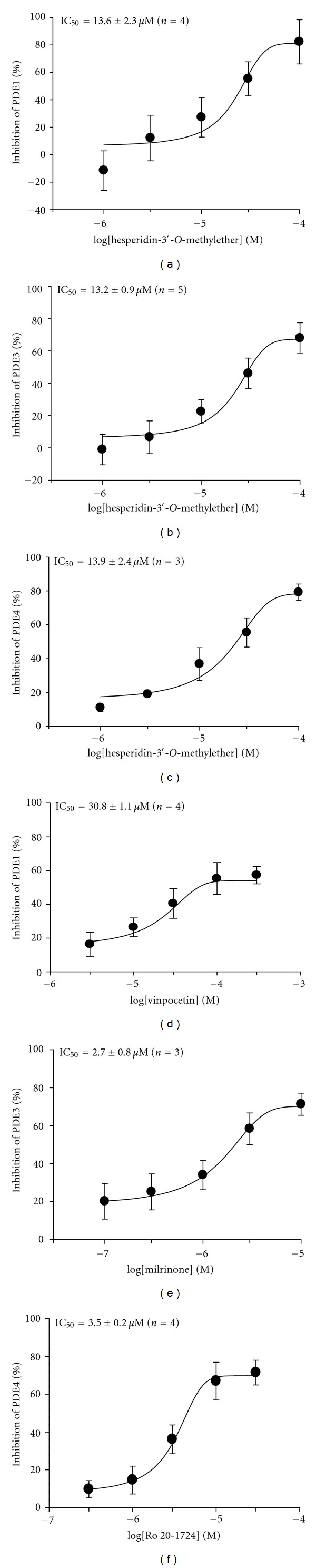
Log concentration-inhibition curves of hesperidin-3′-*O*-methylether (a, b, and c) and reference drugs (d, e, and f) on PDE1 (a, d), PDE3 (b, e), and PDE4 (c, f) activities.

Hesperidin did not inhibit PDE1*∼*5 activities (IC_50_ > 100 *μ*M). Similarly, hesperidin-3′-*O*-methyl ether did not inhibit PDE2 or PDE5 activity (IC_50_ > 100 *μ*M). However, its concentration-dependently inhibited PDE1, PDE3, and PDE4 activities with respective IC_50_ values of 13.6 ± 2.3 *μ*M (*n* = 4), 13.2 ± 0.9 *μ*M (*n* = 5), and 13.9 ± 2.4 *μ*M (*n* = 3) (Figures 3(a), 3(b), and 3(c)). The reference drugs, vinpocetine, milrinone, and Ro 20-1724, also concentration-dependently inhibited these enzymes with respective IC_50_ values of 30.8 ± 1.1 *μ*M (*n* = 4), 2.7 ± 0.8 *μ*M (*n* = 3), and 3.5 ± 0.2 *μ*M (*n* = 4) (Figures 3(d), 3(e), and 3(f)). The IC_50_ values of hesperidin-3′-*O*-methyl ether for PDE1, PDE3, and PDE4 inhibition did not significantly differ from each other.

### 3.2. PDE4_*H*_/PDE4_*L*_ Ratios

**Figure 4:**
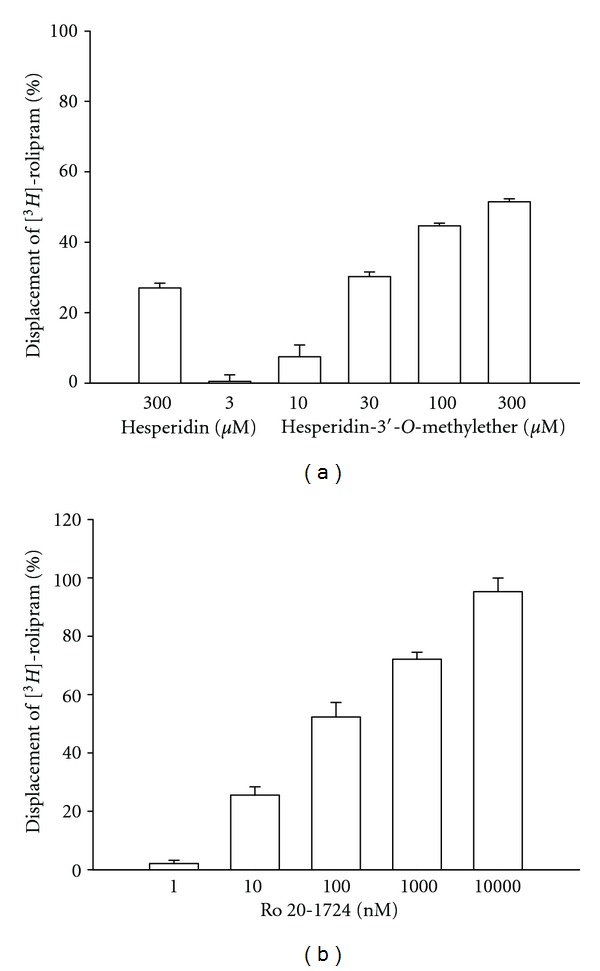
Displacement of [^3^ *H*]-rolipram by hesperidin and hesperidin-3′-*O*-methylether (a) and Ro 20-1724 (b) in high-affinity rolipram binding sites of guinea pig brain particulate. Each value represents the mean ± SEM The experimental number was 6.

Hesperidin (300 *μ*M) displaced 2 nM [^3^ *H*]-rolipram binding on HARBSs of guinea pig brain cell membranes only 27.0 ± 1.4% (Figure 4(a)). In other words, the EC_50_ (PDE4_*H*_) value of hesperidin was >300 *μ*M. However, hesperidin-3′-*O*-methylether (3*∼*300 *μ*M), similar to Ro 20-1724 (3*∼*300 nM), concentration-dependently displaced 2 nM [^3^ *H*]-rolipram binding on HARBSs of guinea pig brain cell membranes (Figures 4(a) and 4(b)). The respective EC_50_ (PDE4_*H*_) values of hesperidin-3′-*O*-methylether, and Ro 20-1724 for displacing [^3^ *H*]-rolipram binding were 218.3 ± 32.1 (*n* = 6) *μ*M and 105.4 ± 13.1 (*n* = 6) nM. While the IC_50_ values of hesperidin, hesperidin-3′-*O*-methylether, and Ro 20-1724 for inhibiting PDE4 catalytic activity were >100, 13.9, and 3.5 *μ*M, respectively, which were taken to be PDE4_*L*_ values. Thus, the PDE4_*H*_/PDE4_*L*_ ratios of hesperidin, hesperidin-3′-*O*-methylether and Ro 20-1724 were 3, 15.7, and 0.03, respectively.

### 3.3. Supression of AHR *In Vivo*

**Figure 5:**
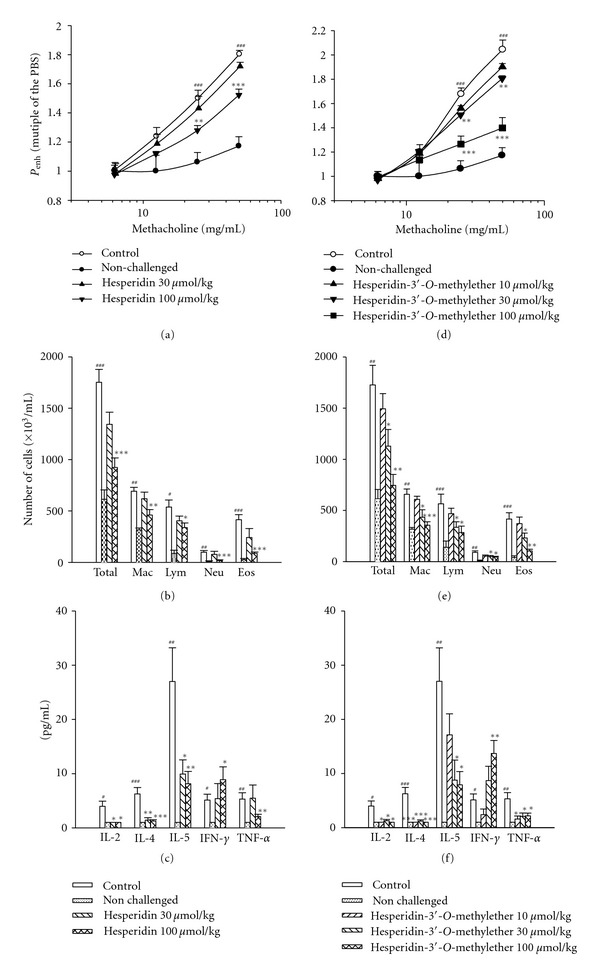
Effects of orally administered hesperidin (a, b, and c) and hesperidin-3′-*O*-methylether (d, e, and f) on the enhanced pause (*P*_enh_, a, d), inflammatory cells (b, e), and cytokines (c, f) in sensitized mice which received aerosolized methacholine (6.25*∼*50 mg/mL) 2 days after the last allergen challenge. ^#^ *P* < 0.05, ^##^ *P* < 0.01, and ^###^ *P* < 0.001, compared to the nonchallenged group. *P < 0.05, **P < 0.01, and ***P < 0.001, compared to the control (vehicle) group. The number of mice in each group was 10. Total, total cells; Mac: macrophages; Lym: lymphocytes; Neu: neutrophils; Eos: eosinophils; IL: interleukin; IF: interferon; TNF: tumor necrosis factor.

*P*_enh_ values at the baseline for the control sensitized and challenged, nonchallenged, and 30, and 100 *μ*mol/kg hesperidin groups were 2.38 ± 0.05, 2.40 ± 0.04, 2.39 ± 0.06, and 2.41 ± 0.04, respectively, and these values did not significantly differ from each other. *P*_enh_ values with PBS nebulization for each group were 2.39 ± 0.06, 2.41 ± 0.04, 2.40 ± 0.05, and 2.39 ± 0.05, respectively, which also did not significantly differ from each other. Administration of nebulized PBS did not affect the *P*_enh_ value of the baseline in each group. However, methacholine (6.25*∼*50 mg/mL) concentration-dependently increased *P*_enh_ values from 1-fold with PBS exposure to 1.80 ± 0.03-fold in control sensitized and challenged mice (Figure 5(a)). *P*_enh_ values of methacholine at 25 and 50 mg/mL in control sensitized and challenged mice were significantly enhanced compared to those in nonchallenged mice. Hesperidin (100 *μ*mol/kg, p.o.) significantly attenuated the enhancement of *P*_enh_ values induced by 25 and 50 mg/mL methacholine (Figure 5(a)).

Similarly, *P*_enh_ values at the baseline for the control sensitized and challenged, nonchallenged, and 10, 30, and 100 *μ*mol/kg hesperidin-3′-*O*-methylether groups were 2.39 ± 0.06, 2.40 ± 0.03, 2.41 ± 0.05, 2.38 ± 0.06, and 2.41 ± 0.05, respectively, and these values did not significantly differ from each other. *P*_enh_ values with PBS nebulization for each group were 2.40 ± 0.04, 2.38 ± 0.05, 2.41 ± 0.06, 2.42 ± 0.05, and 2.39 ± 0.06, respectively, which also did not significantly differ from each other. Administration of nebulized PBS did not affect the *P*_enh_ value of the baseline in each group. Methacholine (6.25*∼*50 mg/mL) also concentration-dependently increased *P*_enh_ values from 1-fold with PBS exposure to 2.04 ± 0.08-fold in control sensitized and challenged mice (Figure 5(d)). *P*_enh_ values of methacholine at 25 and 50 mg/mL in control sensitized and challenged mice were significantly enhanced compared to those in nonchallenged mice. Hesperidin-3′-*O*-methyl ether (30*∼*100 *μ*mol/kg, p.o.) significantly attenuated the enhancement of *P*_enh_ values induced by 25 and 50 mg/mL methacholine (Figure 5(d)).

### 3.4. Supression of Inflammatory Cells in BALF

The numbers of total inflammatory cells, macrophages, lymphocytes, neutrophils, and eosinophils from the BALF of control sensitized and challenged mice significantly increased compared to those of nonchallenged mice (Figure 5(b)). Hesperidin (100 *μ*mol/kg, p.o.) significantly suppressed the increases in numbers of total inflammatory cells, macrophages, lymphocytes, neutrophils, and eosinophils (Figure 5(b)).

The numbers of total inflammatory cells, macrophages, lymphocytes, neutrophils, and eosinophils from the BALF of control sensitized and challenged mice also significantly increased compared to those of nonchallenged mice (Figure 5(e)). Hesperidin-3′-*O*-methylether (30*∼*100 *μ*mol/kg, p.o.) significantly suppressed the increases in numbers of total inflammatory cells, macrophages, lymphocytes, neutrophils, and eosinophils (Figure 5(e)).

### 3.5. Effects on Cytokines in BALF

Compared to those in nonchallenged mice, levels of cytokines, such as IL-2, IL-4, IL-5, IFN-*γ*, and TNF-*α*, in the BALF of control sensitized and challenged mice significantly increased (Figure 5(c)). Hesperidin (30*∼*100 *μ*mol/kg, p.o.) significantly suppressed the increases in levels of IL-2, IL-4, IL-5, and TNF-*α*  with the exception of TNF-*α*  at a dose of 30 *μ*mol/kg (Figure 5(c)). However, hesperidin at a dose of 100 *μ*mol/kg significantly enhanced the level of IFN-*γ* compared to the control (Figure 5(c)).

Compared to those in nonchallenged mice, levels of cytokines, such as IL-2, IL-4, IL-5, IFN-*γ*, and TNF-*α*, in the BALF of control sensitized and challenged mice also significantly increased (Figure 5(f)). Hesperidin-3′-*O*-methylether (10*∼*100 *μ*mol/kg, p.o.) significantly suppressed increases in levels of IL-2, IL-4, IL-5, and TNF-*α*  with the exception of IL-5 at a dose of 10 *μ*mol/kg (Figure 5(f)). However, hesperidin-3′-*O*-methylether at a dose of 100 *μ*mol/kg significantly enhanced the level of IFN-*γ* compared to the control (Figure 5(f)).

### 3.6. Effects on IgG_2a_ and IgE in the Serum and BALF

**Figure 6:**
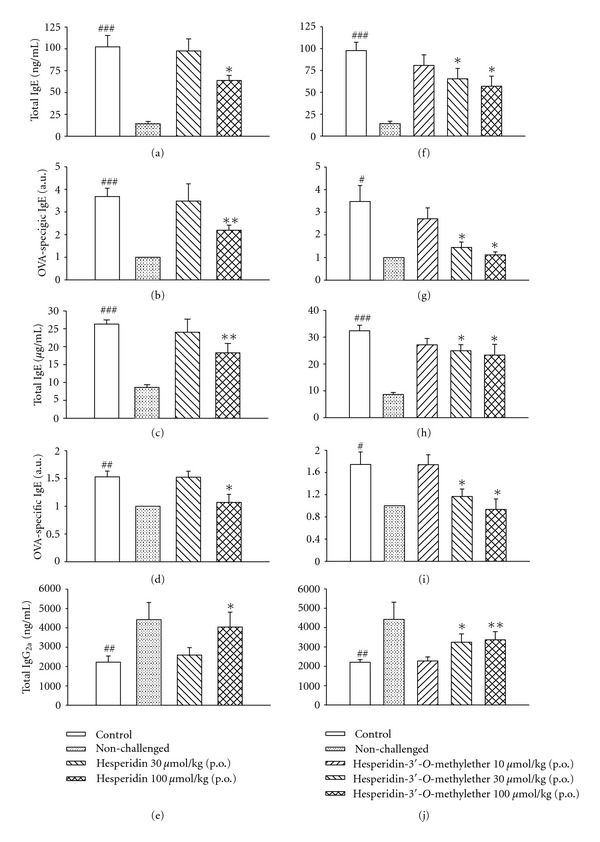
Effects of hesperidin (a–e) and hesperidin-3′-*O*-methylether (f–j) on total IgE (a, c, f, and h) and ovalbumin-specific IgE (b, d, g, and i) levels in bronchial alveolar lavage fluid (a, b, f, and g) and serum (c, d, h, i), and total IgG_2a_ (e, j) levels in serum of sensitized mice which had received aerosolized methacholine (6.25*∼*50 mg/mL) 2 days after primary allergen challenge. ^#^ *P* < 0.05, ^##^ *P* < 0.01, and ^###^ *P* < 0.001, compared to the nonchallenged group. *P < 0.05 and **P < 0.01, compared to the control (vehicle) group. Each value represents the mean ± SEM. The number of mice in each group was 10.

Levels of total and ovalbumin-specific IgE in the BALF and serum of control sensitized and challenged mice were significantly enhanced compared to those of nonchallenged mice. Hesperidin (100 *μ*mol/kg, p.o.) significantly suppressed these enhancements (Figures 6(a), 6(b), 6(c), and 6(d)). The total IgG_2a_ level in the serum of control sensitized and challenged mice was significantly reduced compared to that of nonchallenged mice. Hesperidin (100 *μ*mol/kg, p.o.) significantly reversed this reduction (Figure 6(e)).

Levels of total and ovalbumin-specific IgE in the serum and BALF of control sensitized and challenged mice were also significantly enhanced compared to those of nonchallenged mice. Hesperidin-3′-*O*-methylether (30*∼*100 *μ*mol/kg, p.o.) dose-dependently and significantly suppressed these enhancements (Figures 6(f), 6(g), 6(h), and 6(i)). The total IgG_2a_ level in the serum of control sensitized and challenged mice was significantly reduced compared to that of nonchallenged mice. Hesperidin-3′-*O*-methylether (30*∼*100 *μ*mol/kg, p.o.) dose-dependently and significantly reversed this reduction (Figure 6(j)).

### 3.7. No Effect on Xylazine/Ketamine-Induced Anesthesia

**Figure 7:**
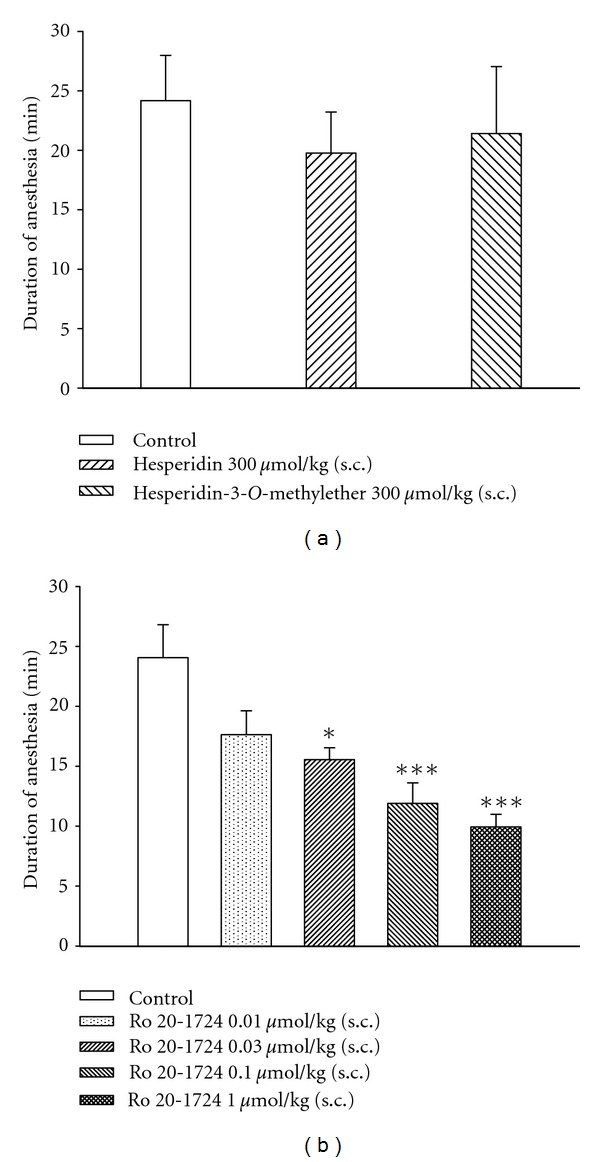
Effects of subcutaneously administered hesperidin and hesperidin-3′-*O*-methylether (a) and Ro 20-1724 (b) on the duration of xylazine (10 mg/kg, i.p.)/ketamine (70 mg/kg, i.p.)-induced anesthesia in mice. Ro 20-1724 was administered 0.25 h and hesperidin and hesperidin-3′-*O*-methylether 1 h before anesthesia. *P < 0.05, ***P < 0.001, compared to the vehicle (control). Each value represents the mean ± SEM. The number of mice in each group was 10.

The durations of xylazine/ketamine-induced anesthesia in control (vehicle) mice for the hesperidin- or hesperidin-3′-*O*-methylether-, and Ro 20-1724-treated groups were 24.2 ± 3.8 (*n* = 10), and 24.1 ± 2.8 min (*n* = 10), respectively. Neither hesperidin nor hesperidin-3′-*O*-methylether (each 300 *μ*mol/kg, s.c.) influenced the duration (Figure 7(a)). In contrast, Ro 20-1724 (0.01*∼*1 *μ*mol/kg, s.c.) dose-dependently shortened the duration and at doses of 0.03*∼*1 *μ*mol/kg (s.c.) significantly shortened the duration (Figure 7(b)).

## 4. Discussion

Allergic asthma is a chronic respiratory disease characterized by AHR, mucus hypersecretion, bronchial inflammation, and elevated IgE levels. Th2 cells, together with other inflammatory cells such as eosinophils, B cells, and mast cells are thought to play critical roles in the initiation, development, and chronicity of this disease [27]. One hypothesis emphasizes an imbalance in Th cell populations favoring expression of Th2 over Th1 cells. Cytokines released from Th2 cells are IL-4, IL-5, IL-6, IL-9, and IL-13, and those from Th1 cells are IL-2, IL-12, IFN-*γ*, and TNF-*α*  [28, 29]. In the present results, hesperidin (100 *μ*mol/kg, p.o.) and hesperidin-3′-*O*-methylether (30*∼*100 *μ*mol/kg, p.o.) significantly attenuated *P*_enh_ values at 25 and 50 mg/mL methacholine (Figures 5(a) and 5(d)) suggesting that it significantly suppresses AHR. At the dose of 30 *μ*mol/kg (p.o.), hesperidin-3′-*O*-methylether, but not hesperidin, significantly suppressed AHR, suggesting that hesperidin-3′-*O*-methylether is more potent than hesperidin in the suppression of AHR. Similarly, hesperidin-3′-*O*-methylether, but not hesperidin, at the dose of 30 *μ*mol/kg (p.o.) significantly suppressed the numbers of all inflammatory cells examined, including total inflammatory cells, macrophages, lymphocytes, neutrophils, and eosinophils in the BALF of mice (Figures 5(b) and 5(e)). Hesperidin-3′-*O*-methylether even at 10 *μ*mol/kg (p.o.) significantly suppressed the level of IL-4 which are released from Th2 cells, although hesperidin at this dose did not perform in this study. However, hesperidin was reported to insignificantly inhibit the level of IL-4 at a dose of 10 mg/kg (16.38 *μ*mol/kg, p.o.) in a similar animal model [13]. Thus it also suggests that hesperidin-3′-*O*-methylether is more potent than hesperidin in the suppression of IL-4, although the levels of IL-5 were suppressed to the same extent by both. Hesperidin-3′-*O*-methylether 10 *μ*mol/kg (p.o.) significantly suppressed the level of IL-2, which are released from Th1 cells, although hesperidin at this dose did not perform in this study. However, hesperidin-3′-*O*-methylether was obviously more potent than hesperidin in inhibition of TNF-*α*  level, suggesting that the former is more potent than the latter in inhibition of Th1 cells. In contrast, the levels of IFN-*γ* were enhanced by both hesperidin and hesperidin-3′-*O*-methylether at 100 *μ*mol/kg (p.o.). These results suggest that hesperidin and hesperidin-3′-*O*-methylether suppress Th2 cells, and partly activate Th1 cells, which ameliorate this imbalance and produce anti-inflammatory effects. Th1 and Th2 cells have been implicated in autoimmune and atopic diseases, respectively [30]. Overall, orally administered hesperidin-3′-*O*-methylether was more potent than hesperidin to have anti-inflammatory effects in this *in vivo* study. The different potency between them may be due to their aglycons, because these two flavanone glycosides will be hydrolyzed by *β*-glucosidase after oral administration [31]. The aglycons of hesperidin-3′-*O*-methylether and hesperidin are hesperetin-3′-*O*-methylether and hesperetin, respectively. We have reported the IC_50_ values of hesperetin-7,3′-*O*-dimethylether and hesperetin for PDE4 inhibition are 3.0 *μ*M [32] and 28.2 *μ*M [10], respectively, although that of hesperetin-3′-*O*-methylether remains unknown. Moreover, in the present results, the IC_50_ values of hesperidin-3′-*O*-methylether for PDE1, 3, and 4 inhibition were 13.6, 13.2, and 13.9 *μ*M, respectively. Thus, that of hesperetin-3′-*O*-methylether for PDE4 inhibition should be less than 13.9 *μ*M, because its the bulky glycosyl residue may be as a steric hindrance for binding to this PDE conformation [33]. By this reason, hesperetin is more active for PDE4 inhibition than hesperidin which was demonstrated to be inactive for PDE1*∼*5 inhibitions in the present results. Hesperidin at 30 *μ*mol/kg significantly suppressed levels of IL-2, IL-4, and IL-5 (Figure 5(c)), and hesperidin-3′-*O*-methylether at 10 *μ*mol/kg significantly suppressed levels of IL-2, IL-4, and TNF-*α*  (Figure 5(f)), although all types of inflammatory cells were unaffected by both at these doses (Figures 5(b) and 5(e)). These inconsistencies may be due to the accuracies of these two measurements, because that cytokines were measured using flow cytometric methods, whereas inflammatory cells were measured using a hemocytometer under light microscopy.

IL-4 and IL-13 were shown to induce AHR in mouse asthma models [34, 35]. IL-4 has three primary effects. First, IL-4 promotes B cell differentiation to plasma cells that secrete antigen-specific IgE antibodies. Second, IL-4 promotes mast cell proliferation. Third, increased IL-4 upregulates endothelial cell expression of adhesion molecules for eosinophils [36]. IL-5 mobilizes and activates eosinophils, leading to the release of a major basic protein, cysteinyl-leukotriene, and eosinophil peroxidase that contribute to tissue damage and AHR [35, 37]. Phosphoinositide 3-kinase *δ* (p110*δ*) was shown to play a crucial role in the development, differentiation, and antigen receptor-induced proliferation of mature B cells [38, 39], and inhibition of p110*δ* attenuates allergic airway inflammation and AHR in a murine asthma model [38, 40]. In addition, IL-4 and IL-13 are important in directing B cell growth, differentiation, and secretion of IgE [41]. However, IFN-*γ* released from Th1 cells preferentially directs B cell switching of IgM to IgG_2a_ and IgG_3_ in mice [42, 43]. In the present results, hesperidin (100 *μ*mol/kg, p.o.) and hesperidin-3′-*O*-methylether (30*∼*100 *μ*mol/kg, p.o.) significantly suppressed total and OVA-specific IgE levels in the serum and BALF, and enhanced the level of total IgG_2a_ in the serum of sensitized and challenged mice, suggesting that both have immunoregulatory effects. At the dose of 30 *μ*mol/kg (p.o.), hesperidin-3′-*O*-methylether, but not hesperidin, significantly suppressed total and OVA-specific IgE levels in the serum and BALF, and enhanced the level of total IgG_2a_ in the serum of sensitized and challenged mice, suggesting that hesperidin-3′-*O*-methylether is also more potent than hesperidin in these immunoregulatory effects. 8-Methoxymethyl-3-isobutyl-1-methylxanthine, a selective PDE1 inhibitor, was reported to block lipopolysaccharide (LPS)-mediated biosynthesis of IL-6, but not to influence the TNF-*α*  level. Furthermore, inhibition of PDE3 by amrinone was reported to abolish the effect of LPS on IL-6, but attenuate TNF-*α*  production. Reversible competitive inhibition of PDE4 by rolipram was reported to exhibit a potent inhibitory effect on IL-6 and a dual, biphasic (excitatory/inhibitory) effect on TNF-*α*  secretion [44]. Selective inhibition of PDE1, 3, and 4 by these three compounds was also reported to exhibit a tendency to augment the translocation of NF-*κ*B_1_ (p50), RelA (p65), RelB (p68), and c-Rel (p75) and associate with upregulating NF-*κ*B transcriptional activity [45]. These immunopharmacological effects may be found in the administration of hesperidin-3′-*O*-methylether with a similar extent for PDE1, 3, and 4 inhibition.

Selective PDE4 inhibitors specifically prevent the hydrolysis of cAMP, a 3′,5′-cyclic nucleotide, and therefore have broad anti-inflammatory effects such as inhibition of cell trafficking and of cytokine and chemokine release from inflammatory cells. The increased cAMP levels induced by these selective PDE4 inhibitors subsequently activate cAMP-dependent protein kinase which may phosphorylate and inhibit myosin light-chain kinase, thus inhibiting contractions [46]. The precise mechanism through which relaxation is produced by this second-messenger pathway is not known, but it may result from decreased intracellular Ca^2+^ ([Ca^2+^]_*i*_). The decrease in  [Ca^2+^]_*i*_  may be due to reduced influx of Ca^2+^, enhanced Ca^2+^ uptake into the sarcoplasmic reticula, or enhanced Ca^2+^extrusion through cell membranes [46]. Thus hesperidin-3′-*O*-methylether may have bronchodilatory effects and may be useful in treating COPD.

In the present *in vitro* studies, the PDE4_*H*_/PDE4_*L*_ ratios of hesperidin and hesperidin-3′-*O*-methylether were calculated to be 3 and 15.7, respectively. However, neither hesperidin nor hesperidin-3′-*O*-methylether administered (s.c.) influenced xylazine/ketamine-induced anesthesia. This may be due to the administration route, but administered (s.c.) hesperetin, an aglycon of hesperidin hydrolyzed after oral administration, was reported to not influence xylazine/ketamine-induced anesthesia [47]. Nevertheless, Ro 20-1724, a selective PDE4 inhibitor, reversed the anesthesia. The reversing effect may occur through presynaptic *α*_2_-adrenoceptor inhibition [48], because MK-912, an *α*_2_-adrenoceptor antagonist, was reported to reverse xylazine/ketamine-induced anesthesia in rats [8] and trigger vomiting in ferrets [48]. In contrast, clonidine, an *α*_2_-adrenoceptor agonist, prevented emesis induced by PDE4 inhibitors in ferrets [48]. The present results also suggest that hesperidin and hesperidin-3′-*O*-methylether may have few or no adverse effects, such as nausea, vomiting, and gastric hypersecretion. In addition, PDE4 subtypes (A*∼*D) may be considered for drug development of new PDE4 inhibitors. PDE4D inhibition in nontarget tissues promotes emesis, since PDE4D knock-out mice showed reduction of xylazine/ketamine-triggered anesthesia which is used as a surrogate marker for emesis in mice, a nonvomiting species [9]. In contrast to PDE4D, selective inhibition of PDE4A and/or PDE4B in proinflammatory and immune cells is believed to evoke the therapeutically desired effects of these drugs [49]. Thus, hesperidin-3′-*O*-methylether did not influence xylazine/ketamine-induced anesthesia may be due to its selectivity for PDE4A and/or PDE4B inhibition(s). However, whether hesperidin-3′-*O*-methylether selectively inhibits the PDE4 subtype needs to be further investigated.

In conclusion, hesperidin-3′-*O*-methylether may be more potent than hesperidin in anti-inflammatory and immunoregulatory effects, including suppression of AHR, and reduced expressions of inflammatory cells and cytokines in the murine model of allergic asthma. In addition, neither hesperidin nor hesperidin-3′-*O*-methylether influenced xylazine/ketamine-induced anesthesia, suggesting that they have few or no emetic effect. Thus, hesperidin-3′-*O*-methylether may have more potential than hesperidin for use in treating allergic asthma and COPD.

## Abbreviations

cAMP: Adenosine 3′,5′ cyclic monophosphate
cGMP: Guanosine 3′,5′ cyclic monophosphate
COPD: Chronic obstructive pulmonary disease
DMSO: Dimethyl sulfoxide
EDTA: Ethylenediaminetetraacetic acid
HARBSs: High-affinity rolipram-binding sites
IFN: Interferon
Ig: Immunoglobulin
IL: Interleukin
PBS: Phosphate-buffered saline
PDE: Phosphodiesterase
PDE4_*H*_: High affinity for PDE4
PDE4_*L*_: Low affinity for PDE4
*P*_enh_: Enhanced pause
PMSF: Phenylmethanesulfonyl fluoride
Ro20-1724: 4-(3-butoxy-4-methoxybenzyl)-2-imidazolidinone
TCM: Traditional Chinese medicine
Th: T-helper
TNF: Tumor necrosis factor.
